# A systematic review of attachment interventions for people with intellectual disability and their caregivers

**DOI:** 10.1177/17446295241289734

**Published:** 2024-10-04

**Authors:** Ruby Ramsden, Emily Reeves, Eve Whitwell, Nicola Lane, Helen K Fletcher

**Affiliations:** Oxford Institute for Clinical Psychology Research and Training, 6396 University of Oxford, UK; Oxford Institute for Clinical Psychology Research and Training, 6396 University of Oxford, UK; Oxford Institute for Clinical Psychology Research and Training, 6396 University of Oxford, UK; Oxford Institute for Clinical Psychology Research and Training, 6396 University of Oxford, UK; 7482 Hertfordshire Partnership University NHS Foundation Trust, UK

**Keywords:** learning disability, intellectual disability, attachment theory, attachment interventions, relationships

## Abstract

**Objective:**

The current mixed-methods systematic review evaluated available literature to find out which attachment-based interventions have been implemented for people with intellectual disability and whether they are efficacious and acceptable.

**Methods:**

Five databases were searched (in July 2023 and April 2024), using terms related to intellectual disability and attachment-based interventions. The search yielded 793 papers; 15 papers (13 studies) met inclusion criteria. Relevant data was extracted from each study. Paper quality was appraised using the Mixed Methods Appraisal Tool. Findings were synthesised in an integrative review.

**Results:**

Of the included studies, 7 had people with intellectual disability as participants and 6 had their caregivers. Interventions included education, psychotherapy, technology assisted therapy, video interaction guidance/feedback and circle of security. Research methods varied.

**Conclusions:**

Evidence for efficacy and acceptability of interventions was mixed but promising. Most studies had limited generalisability. Therefore, further research is required. Pre-registration with PROSPERO [351287].

## Introduction

### Intellectual disabilities and attachment theory

Intellectual disability is characterised by significant difficulties with intellectual functioning (intelligence quotient (IQ) <70) and adaptive functioning (i.e. ability to complete developmentally appropriate daily activities) with childhood onset (American Psychiatric Association, 2013; BPS, 2015). Intellectual disabilities are categorised based on level of functioning – as mild, moderate or severe and profound (BPS, 2015). People with intellectual disability also have increased prevalence of diagnoses of Autism Spectrum Disorder (ASD), physical disabilities and sensory disabilities, compared to people without intellectual disability (Department of Health, 2001; NHS England and NHS, 2019). Furthermore, they are thought to have increased difficulties with forming and maintaining attachment relationships (Hamadi and Fletcher, 2021).

According to attachment theory, a parent/caregiver’s sensitivity (ability to perceive and accurately interpret communication signals) and responsiveness to their infant’s needs influences the infant’s attachment strategies (how they form and maintain relationships with others), view of themselves and others, as well as their regulation of emotions and behaviour throughout the life span (Bowlby, 1969; Ainsworth et al., 1978; Bowlby, 1977; Groh et al., 2017). Infants who experience care as consistent and responsive are thought to develop ‘secure’ attachment strategies (they are able to seek support from others and self-regulate their emotions), whereas infants who experience care as inconsistent or inadequate develop insecure attachment strategies (sometimes categorised as ‘anxious/ambivalent’ or ‘avoidant’). Those who experience care as fearful or frightening develop disorganised attachment strategies (Main and Solomon, 1990; Ainsworth et al., 1978). All attachment strategies are considered adaptive for the infant’s environment.

People with intellectual disability have been found to have increased prevalence of insecure and disorganised attachment strategies compared to secure, in both clinical and non-clinical populations (Hamadi and Fletcher, 2021; Bateman et al., 2023). See Cassidy (2016) and Solomon and George (2016) for further understanding of early attachment theory and categorisation. The current paper considers a broad conceptualisation of attachment theory, attachment-related behaviour (any behaviour guided towards attaining and maintaining the availability of an attachment figure) and the implications for care-receiving and care-giving attachment relationships for people with intellectual disability, for reasons discussed below (Verhage et al., 2023; Schuengel et al., 2013).

There are a number of reasons why people with intellectual disability may experience increased difficulties with attachment relationships (Fletcher et al., 2016). For example, parents may be less likely to consistently recognise and meet needs of children with intellectual disability, due to their difficulty communicating/expressing them – requiring additional sensitivity and attunement (Giltaij et al., 2015; Schuengel and Janssen, 2006). Additionally, parents of children with intellectual disability report increased distress and mental health (MH) difficulties, which can impact interactions and attachment (Singer, 2006). The role of parental grief for an imagined ‘healthy child’ is suggested to impact attunement to a child’s needs (Fletcher, 2016; Atkinson et al., 1999). Furthermore, people with intellectual disability are at increased risk of physical and psychological abuse, which is associated with insecure and fearful attachment strategies (Wright, 2013). People with intellectual disability also often require additional support, i.e. from family, professional carers (PCs) or residential care, meaning they may experience a high number of, and changes to, caregiving and attachment figures across the lifespan (Fletcher et al., 2016). This poses additional challenges to the development and maintenance of secure attachment relationships when individuals are reliant on multiple carers.

People with intellectual disability may sometimes express attachment needs through distressed behaviour, sometimes referred to as “challenging behaviour” (CB) (Skelly, 2016). This can be distressing for caregivers, both familial and professional. Other attachment-related behaviour which can be experienced as ‘challenging’ for PCs includes people with intellectual disability becoming ‘overly fond’ of them (expressed by them following PCs around or becoming upset when they leave) (Larson et al., 2011). It is important to consider how caregivers respond to distress, CB, and attachment-behaviours and how they develop and maintain relationships with people with intellectual disability, as this may impact future distress, communication and relationships. Variations in attachment behaviour of people with intellectual disability has been found to be partly explained by differences amongst professional caregivers (De Schipper and Schuengel, 2010), confirming the individual nature of the person’s attachment relationship to each caregiver.

Additional measurement sensitivity and considerations may be needed to study attachment and related concepts in people with intellectual disability, particularly those with significant communication difficulties (Walker et al., 2016). Walker et al. (2016) argues further investigation and validation of attachment specific measures for people with intellectual disability is warranted due to a lack of established measures. However, the effectiveness of attachment-based interventions may be indicated through varied different outcomes, such as reduced distress, reduced distressed behaviour, improved emotional wellbeing and/or increased parental sensitivity or attunement (Rinaldi et al., 2022; O'Hara et al., 2019; Kennedy et al., 2011; Birdsey et al., 2022; Hodes et al., 2017; 2018; Ainsworth et al., 1974; Skelly, 2016).

### Attachment interventions

Attachment difficulties and insecurity are associated with MH difficulties, increased distress and distressed behaviour (Conradi and de Jonge, 2009; Pielage et al., 2005), which are all prevalent within intellectual disability populations (Mullen, 2018). Due to increased risk, it is important appropriate support is implemented for people with intellectual disability and their caregivers. Furthermore, as parents with mild intellectual disability are over-represented within child protection services, and their children disproportionately adopted rather than provided family support (Booth et al., 2005), interventions supporting parent-child relationships are important.

Attachment interventions aim to improve the relationship and sensitivity between caregivers and care-receivers, in the hope of supporting emotional wellbeing and functioning (BPS, 2017; Steele and Steele, 2017). Although most attachment-based interventions were not developed for people with intellectual disability, they are considered transferable (Fletcher and Gallichan, 2016). Interventions finding promising results (in people without intellectual disability) include video-feedback, video-interaction guidance (VIG), the circle of security (COS), and child-parent psychotherapy (Kennedy et al., 2011; Yaholkoski et al., 2016; Cicchetti et al., 2006; Van Ijzendoorn et al., 2023). Alexander et al.'s (2023) review of attachment interventions for children with disabilities or developmental delay, found emerging literature indicated early attachment interventions may effectively increase attachment security in that population. Research evaluating attachment-based interventions for people with intellectual disability and/or borderline intellectual disability is also beginning to emerge, with promising outcomes (Hodes et al., 2017; Hofstra et al., 2023). Furthermore, increasing awareness of attachment theory has been suggested helpful for increasing sensitivity of people supporting people with intellectual disability (Schuengel et al., 2013).

### Relevance of study

Hamadi and Fletcher's (2021) systematic review concluded further evaluation of the efficacy of attachment-based interventions for people with intellectual disability is required, to help understand their impact on reducing distress and MH difficulties. Further development and availability of effective attachment-interventions may help to increase independence, quality of life (QoL) and wellbeing for people with intellectual disability. There is currently no systematic review exploring efficacy/effectiveness and acceptability (whether consumers or participants experience an intervention as satisfactory i.e. in terms of content, comfort, delivery or credibility (Proctor et al., 2011)) of attachment-based interventions for people with intellectual disability specifically. The current mixed-methods review aims to critically evaluate available literature and answer the following questions:

• What type of attachment-based psychological interventions have been implemented for people with intellectual disability across the lifespan?
• Are attachment-focussed psychological interventions efficacious and acceptable for people with intellectual disability and their support networks/carers?

## Method

A mixed-methods integrative review was conducted to appraise and synthesise quantitative, qualitative and mixed-methods research. An integrative approach was taken to help provide a general overview of the current attachment-based intervention literature (both quantitative and qualitative), given the topic is potentially broad, literature sparse and research methods varied (Noyes et al., 2019). Findings of qualitative and quantitative data were integrated. The Preferred Reporting Items for Systematic Reviews and Meta-Analyses (PRISMA) reporting guidelines were followed (Page et al., 2021; Shamseer et al., 2015; Moher et al., 2009). The findings between and within the studies were explored within the integrative synthesis. The review was pre-registered with PROSPERO [351287].

### Search strategy

A systematic review was conducted using five electronic databases: PsychINFO, Medline, CINAHL, Scopus and Cochrane library. Google Scholar was used for additional scoping to ensure search completeness and reference lists of included studies hand-reviewed. Searches were conducted in July 2023 and updated in April 2024.

#### Search terms

The search terms aimed to capture intellectual disability and attachment-based intervention studies (see Appendix 2).

#### Eligibility criteria

Inclusion criteria:

• Qualitative, quantitative and mixed-method research studies – evaluating completed attachment-based interventions for people with intellectual disability (mild – severe and profound) and their caregivers
  ○ Attachment-based interventions: individual or group-based interventions aimed at improving attachment, parent-child/carer-people with intellectual disability interactions or relationships, or factors that support secure attachment
• Participants of any age, who completed an attachment-based intervention for people with intellectual disability - including people with intellectual disability, parents/guardians or PCs
• Research undertaken in any setting
• Papers published any year

Exclusion criteria:

• Not a completed research study (i.e., theoretical papers, systematic reviews, meta-analyses, reflective case studies, books, or pre-registrations)
• Not written in English
• Studies where not all participants have mild – severe and profound intellectual disability (i.e. some have developmental delay or borderline intellectual disability), or are a caregiver of people with intellectual disability (i.e. participants are intervention facilitators)

### Study selection

Identified papers were extracted into Endnote reference management software for screening. Duplicate papers were removed. All titles and abstracts were reviewed according to inclusion and exclusion criteria, and a random 20% sample of papers were double screened by an independent reviewer. Cohen’s kappa statistic, a measure of inter-rater reliability, was used to determine scoring agreement amongst the two researchers (k=0.93). Discrepancies (n=2) were discussed and resolved. As inter-rater reliability was high, remaining abstracts and titles were screened by the lead author only.

The remaining papers underwent full-text screening by the lead author and 25% were additionally full-text screened by the independent reviewer (k=0.81). Discrepancies (n=1) were discussed and resolved. As inter-rater reliability was high, remaining texts were screened by the lead author only.

**Figure 1.**
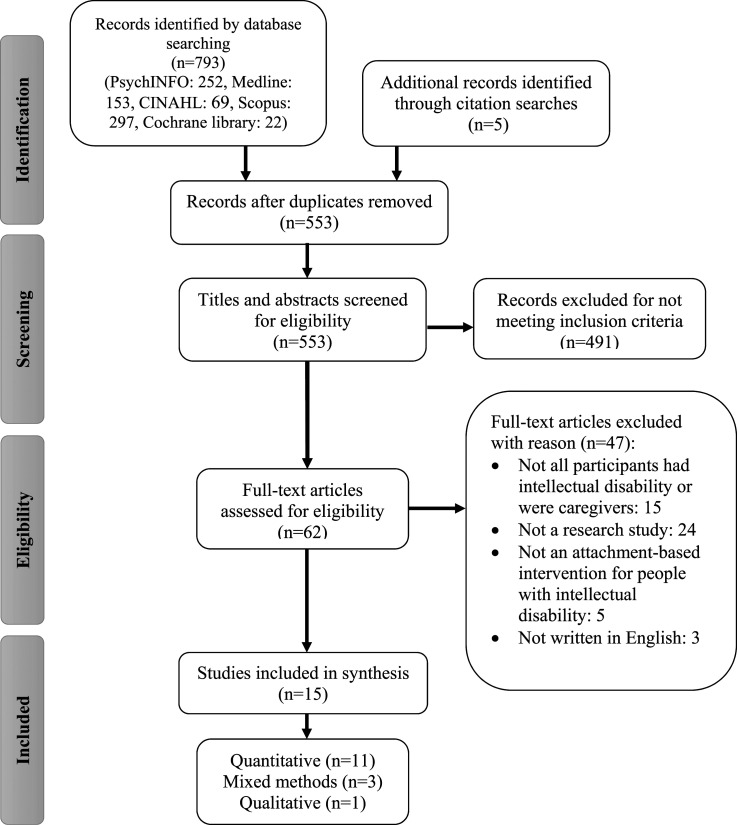
PRISMA flow chart – study identification and selection.

The selection process is summarised in the PRISMA flowchart (see Figure 1). This figure illustrates the number of papers found (793) and included/excluded at each stage, including reasons for exclusion at full-text screening. In total, 15 papers were eligible for the current review. These figures include the updated search in April 2024, which yielded 27 additional documents (three of which were duplicates), none of which met inclusion criteria for the current review.

#### Data extraction and quality assessment

A data extraction tool was created on Microsoft Excel to increase reliability. Data regarding study characteristics was extracted from each paper meeting inclusion criteria. An independent reviewer also extracted data from 50% of studies. Any discrepancies (n=1) were resolved via discussion. Extracted data was organised by type of participant group – people with intellectual disability (see Table 2) and their caregivers (see Table 3). Quantitative, qualitative and mixed-methods studies are presented within the same tables, with findings synthesised in the synthesis/results section.

The Mixed Methods Appraisal Tool (MMAT) was used to assess quality of each study’s methodology (Hong et al., 2018). This tool consists of two optional screening questions (included in the current review), to determine if the study is empirical research, and a total of 25 appraisal questions (Hong et al., 2018). It can appraise five different types of methodology: qualitative, RCTs, non-randomised, quantitative descriptive and mixed-methods. Each methodology has 5 appraisal questions (covering criteria most relevant) (Hong et al., 2018). Mixed-methods studies are appraised with 15 questions (five each for the qualitative, quantitative and mixed-method element). Questions can be answered “yes”, “no” or “can’t tell”. Case-studies and case-series were appraised using the ‘quantitative descriptive’ criteria as results of these studies were predominantly described and presented for individual participants only. The tool does not provide cut offs or scores to define paper quality - the authors recommend sharing scoring for each relevant item, as this is more informative. However, descriptors can be used to illustrate percentage of quality criteria met, if accompanied by further detail (Hong, 2020). See Appendix 1 for quality questions and criteria.

The lead author and independent reviewer independently rated 53% of included papers using the MMAT. Initial inter-rater reliability was high (K=0.82), with reviewers having 93% agreement. Reviewers met to discuss scoring and associated discrepancies (n=4). They discussed each item until discrepancies were resolved and a standardised way of interpreting the item was agreed. As inter-rater agreement was high, only the lead author appraised the remaining papers.

The current review examined papers with different methodologies and participant groups. Findings were synthesised using an integrative synthesis and organised according to participant groups: people with intellectual disability and their caregivers. Quality appraisals of each study were included within the synthesis, to summarise research strengths/limitations.

## Results/synthesis

The current review included 15 papers. Multiple reports of the same research study were collated and appraised as a single study (Higgins et al., 2023). Therefore, the current review included 13 studies (total participants across studies: N=265). All scored “yes” on the screening questions and were deemed empirical research (Hong et al., 2018). Attachment interventions were available across all levels of intellectual disability and the lifespan. Included interventions were education, video-feedback/VIG, psychotherapy, COS and technology assisted therapy for social anxiety (SA). Research methodologies, data collection, and outcome measures varied. Quality of studies varied from meeting 20% of quality criteria to 100%. See Table 1 for quality ratings.

**Table 1.** Quality ratings using MMAT.

Author (year)	1.1	1.2	1.3	1.4	1.5	2.1	2.2	2.3	2.4	2.5	3.1	3.2	3.3	3.4	3.5	4.1	4.2	4.3	4.4	4.5	5.1	5.2	5.3	5.4	5.5	Quality criteria met
Birdsey et al. (2022)	Y	Y	Y	Y	Y											Y	Y	Y	Y	CT	Y	Y	Y	Y	CT	****
Damen et al. (2011) & Schuengel et al. (2012)																Y	Y	Y	N	Y						****
Hoffman et al. (2019)																Y	Y	N	CT	Y						***
Johnson et al. (2017)	Y	Y	Y	Y	Y											Y	CT	N	CT	Y	Y	Y	N	N	N	**
Jonker et al. (2015)																Y	Y	N	CT	Y						***
McInnis (2016)																Y	Y	N	N	N						**
Muddle et al (2022)	Y	Y	Y	Y	Y																					*****
Pearson et al. (2019)											Y	Y	Y	Y	Y											*****
Pethica and Bigham (2018)																Y	Y	N	Y	N						***
Sterkenburg et al. (2008a) & Schuengel et al. (2009)																Y	Y	Y	N	Y						****
Sterkenburg et al. (2008b)																Y	Y	Y	Y	Y						*****
Vandesande et al. (2023)	Y	CT	N	N	N						Y	N	N	N	Y						N	Y	CT	N	N	*
Wingerden et al. (2019)						CT	Y	N	CT	N																*

NB: Key: yes (Y), no (N) and can’t tell (CT).

*****means 100% quality criteria met, **** means 80% quality criteria met, *** means 60% quality criteria met, ** means 40% quality criteria met,* means 20% quality criteria met.

Most studies (12/13) conceptualised attachment as the emotional/affectional bond between caregiver and care-receivers, which helps facilitate emotional coping and exploration of the world (Bowlby, 1969; Ainsworth et al., 1974; Bowlby, 1977; Bowlby, 1988). Caregiver attunement and sensitivity were recognised as facilitators of secure attachments (Ainsworth et al., 1974). Eight studies included people with intellectual disability as direct participants and six included caregivers as direct participants. Results are summarised within these two categories and relevant interventions for each.

### People with intellectual disability

Various interventions and methods were conducted with people with intellectual disability. All used quantitative data and most used case-study or case-series designs (6/8). Participants varied from 10-years-old to 56-years-old. Four studies included participants with comorbid visual impairment (VI), and one focused on a parent with intellectual disability (it was not reported that their children had intellectual disability). All seven studies explored efficacy/effectiveness, and one also explored acceptability (see Table 2 for further detail).

**Table 2.** Extracted data from people with intellectual disability studies.

Author (year)	Study design	Attachment intervention	Sample size	Summary of participant traits	Data collection methods	Main outcome measures	Main findings
Hoffman et al. (2019)	Case-series (experimental)	Technology assisted therapy for social anxiety (TTSA)	N=6	Aged 27-56. Mild to moderate intellectual disability. Additional needs: blind or visual impairment (VI) & social anxiety (SA).	Observations (by professional carers [PCs]), questionnaire (self-report)	Adult behaviour checklist (ABCL); brief symptom inventory (BSI); psychopathology inventory (PIMRA) anxiety subscales; intellectual disability quality of life (IDQOL); frequency of messages.	**Efficacy:** Anxiety scale scores sig. reduced (self and carer-report). Anxious and angry messages, anxious behaviour and CB sig. reduced. Psychological functioning and QoL increased sig.
Jonker et al. (2015)	Single case-study (experimental)	TTSA	N=1	Age: 27. Moderate intellectual disability. Additional needs: VI & SA.	Observations (by PCs), questionnaires (self-report and carer report)	ABCL; BSI; PIMRA anxiety subscales; residential daily observation lists; frequency of messages; social validity questionnaire.	**Efficacy:** Anxious and angry messages sig. reduced. ABCL and BSI indicated significant anxiety reductions, whilst PIMRA did not. The ABCL aggression subscale showed CB did not sig. change. PCs reported reduction in frequency and intensity of anxious and CB. **Acceptability:** Practicality and effectiveness were rated lower post-intervention by PCs. Client rated intervention higher post-intervention, but differences were not significant.
McInnis (2016)	Single case-study	Disability psychotherapy	N=1	Age: 28. Mild intellectual disability.	Observations (by PCs), projective techniques, questionnaires (self & carer-report)	The Frankish Model (FM) of emotional development (observations); object relations technique (ORT) and house tree person (HTP) – projective techniques; target problem behaviour recording sheet; BSI; Manchester attachment scale (MAST).	**Efficacy:** The FM tool found improvements in wellbeing, development and functioning. Various ORT and HTP changes were reported. Problem behaviour reduced from session 22 (first data collection) and ceased session 88. BSI had minimal change. MAST scores were not reported, therapist stated baseline assessment reflected insecure attachment and post-intervention reflected secure.
Pearson et al. (2019)	Non-randomized control trial	Education	N=25	Aged: 16-22. Mild to moderate intellectual disability.	Questionnaire (self-report, via structured interview)	Parent-child questionnaire (created for study, assessing attachment knowledge).	**Efficacy:** Knowledge sig. increased. Gender, age and IQ were not sig. associated with knowledge increase.
Pethica & Bigham (2018)	Single case-study	Video interaction guidance	N=1	Parent. Mild intellectual disability.	Questionnaire (self-report), observations (by researchers)	Self-assessment of parenting (using visual scale); observations of sensitive interactions (during recordings); verbal feedback from professionals.	**Efficacy:** Participant reported feeling more confident she was able to be listened to by her children. Sensitive interactions increased. Professionals reported they appeared more confident in parenting interactions. The children were removed from the child protection register towards the end of the intervention.
Sterkenburg et al. (2008a) & Schuengel et al. (2009)	Case-series (experimental)	Integrative attachment-psychotherapy and behaviour modification (BM)	N=6	Aged: 10-17. Severe intellectual disability. Additional needs: blind or VI.	Observations (by PCs and researchers)	Severe challenging behaviour consensus protocol (CEP); challenging behaviour scale for people with intellectual disability (SGZ); residential observation lists of challenging behaviour (CB); frequency of target behaviour (in therapy videos). Physiological arousal indicators.	**Efficacy:** CEP scores sig. reduced. SGZ changes were not significant. Frequency of CB reduced (for those with reliable data). Target adaptive behaviour sig. increased for attachment therapist. Reductions in target CB were not sig. different between therapists. Physical indicators had mixed results across participants.
Sterkenburg et al. (2008b)	Single case-study (experimental)	Integrative attachment-psychotherapy and BM	N=1	Age: 17. Severe intellectual disability. Additional needs: blind.	Observations (by PCs and researchers)	Residential observation lists (of CB); frequency of target behaviour (during therapy videos); attachment behaviour observations; physiological arousal indicators.	**Efficacy** CB sig. reduced. BM conducted by the attachment-therapist led to sig. more adaptive target behaviour than the control therapist. Physical indicators had mixed results. Proximity seeking and avoidant attachment behaviours were demonstrated sig. more with the attachment therapist (contact maintenance and resistance were not).

#### Education

Education interventions aim to increase knowledge of attachment and relationship principals, in hope of subsequently increasing attachment-related behaviours. Only one study (Pearson et al., 2019) used education. Pearson et al. (2019) evaluated an attachment-education DVD intervention for young adults with intellectual disability (who were not current or expectant parents), to explore effectiveness in improving attachment knowledge (regarding parent-baby relationships). It found statistically significant increases in knowledge, providing evidence for efficacy.

#### Quality appraisal

Pearson et al.'s (2019) non-randomised study met 100% quality criteria, providing preliminary high-quality evidence that attachment-education interventions may be efficacious and acceptable for increasing attachment-knowledge. Use of follow-up evaluation is a strength. Despite promising results, the study does not determine whether increased knowledge would increase attachment-related behaviours. As participants were not parents, conclusions cannot be drawn regarding parent-child relationships. However, the intervention could be a helpful early-intervention for future parents. Further study is needed to confirm findings, particularly with randomised design, using parents who have intellectual disability and using behaviour change measures.

#### Psychotherapy

Three studies (Sterkenburg et al., 2008a; 2008b; McInnis, 2016; Schuengel et al., 2009), using case-study and case-series design evaluated attachment-based psychotherapy adapted for people with intellectual disability.

Sterkenburg et al.'s (2008a) study (also reported in Schuengel et al. (2009)), and Sterkenburg et al.'s (2008b) evaluated integrative attachment-based behaviour modification (BM) via experimental case-study and case-series. Intervention incorporated attachment-focussed psychotherapy (the therapist built an attachment-relationship with participants through sensitive and positive responses) and BM to reduce distressed behaviour. Both studies found reductions in emotional and behavioural difficulties, suggesting the intervention is efficacious. They both found BM conducted by the attachment-therapist led to significantly more adaptive target behaviour than the control therapist, suggesting attachment-based BM is more advantageous than standard.

McInnis (2016) evaluated ‘disability psychotherapy’ which used psychodynamic and counselling approaches to promote psychological wellbeing and attachment security (Frankish, 2013). Findings suggested the intervention was efficacious as it reduced emotional difficulties and distressed behaviour.

#### Quality appraisal

Sterkenburg et al. (2008a) and Schuengel et al. (2009) met 80% quality criteria and Sterkenburg et al. (2008b) met 100%. Overall quality was high for the chosen methodology, particularly use of valid and/or reliable measures. However, Sterkenburg et al. (2008a) omitted data regarding CB for 2/6 participants as reports were inconsistent/unreliable, which could have biased results. Sterkenburg et al. (2008b) was strengthened by including an attachment-based observation tool to assess impact on attachment behaviours, although not validated, it had acceptable inter-rater reliability. Both studies are also praised for their use of experimental design, a control therapist and use of independent observers blinded to the intervention conditions. However, although they stated more attachment-behaviours were demonstrated with the attachment-therapist, as proximity-seeking and avoidant attachment behaviours significantly increased compared to the control therapist, it could be argued these findings are contradictory and suggestive of an insecure attachment relationship.

McInnis' (2016) explorative case-study met 40% quality criteria. Although they adapted measures for the participant’s cognitive abilities (i.e. using drawings to illustrate Brief Symptom Inventory [BSI] answers), which is a clinical strength, it may have impacted validity and reliability. Further measurement issues included: no report of projective technique psychometric properties, introducing the behaviour observation sheet in session 22, and the attachment scale being completed retrospectively. These factors, with the limited sample size and lack of statistical analysis, suggest conclusions must be interpreted with caution.

These studies add to the limited attachment psychotherapy literature for people with intellectual disability. Studies by Sterkenburg et al. (2008a; 2008b) provide preliminary evidence for efficacy of attachment-based BM reducing distressed behaviour. However, they are limited by their case-study and case-series design which lack generalisability and follow-up. McInnis' (2016) evidence, although positive, is limited by poor appraisal quality rating. Further evidence is needed to support efficacy findings, and research which also explores whether participants find the intervention acceptable.

#### Technology assisted therapy for separation anxiety (SA)

Jonker et al. (2015) and Hoffman et al. (2019) evaluated effectiveness of technology assisted therapy for SA for intellectual disability and visual impairment (VI), using experimental case-study and case-series designs. They aimed to reduce SA and teach person permanence via a digital application to contact caregivers whilst apart. It involved participants sending pre-set messages about their mood, i.e. “I am sad” and caregivers responding acknowledging this i.e. “you are sad”. All exchanged messages were discussed when reunited, based on an attachment-based protocol (Marvin et al., 2002). Both studies included observations, questionnaires and frequency of messages. They also introduced participants to the technology (iPhone) prior to the main intervention (and measured the impact), to account for possible confounding effects i.e. in case the introduction of the phone, rather than the intervention, caused the positive effects. Results suggest the intervention may be efficacious at reducing anxiety and distressed behaviour. Jonker et al. (2015) also explored acceptability of the intervention by incorporating social validity questionnaires for participants and caregivers. Acceptability outcomes indicated participants and caregivers were generally positive about the instrument at completion. However, caregivers rated practicality and effectiveness of device lower post-intervention, suggesting it was not as effective or practical as expected. In future research, it would be helpful to explore why that was (i.e. by using open ended questions/interview).

#### Quality appraisal

Jonker et al. (2015) and Hoffman et al. (2019) both met 60% quality criteria for their case-study and case-series design. The novel approach of using adapted technology is innovative. Both studies used valid and reliable measures, but neither used a SA measure despite this being the intervention focus and suggesting it significantly reduced it (Hoffman et al., 2019) – this is a significant limitation. There are limited outcome measures validated for people with intellectual disability and authors claimed none existed for SA, however, aims and conclusions should have been adapted accordingly (e.g. stating the intervention aimed to reduce anxiety). Use of both carer and SU reports is a strength, and inclusion of an adapted SU validity scale (using images) is a clinical strength, although it may impact reliability and validity, particularly as it was the only study to explore acceptability for participants with an intellectual disability.

Despite appraised strengths, it was not possible to determine risk of non-response bias, as both studies failed to report how much data was imputed. Overall, the studies provide preliminary evidence for the intervention being efficacious, with some evidence regarding acceptability. However, further research is needed, particularly non-randomised and randomised group studies, to address limited generalisability and methodological issues/reporting.

#### Video-feedback/interaction guidance

National Institute for Clinical Excellence (NICE) (2016) recommended video-feedback (or ‘video interaction guidance’ [VIG]) to improve parent-child attachment. It aims to increase parental sensitivity (recognition and response to child needs) (Ainsworth et al., 1974; Kennedy et al., 2011). Parents/caregivers are supported to reflect on videos of interactions with their baby/child, and feedback is used to reinforce sensitivity.

Pethica and Bigham's (2018) VIG case-study used observations and self-reported rating of parenting (via a visual scale). It incorporated adaptations for people with intellectual disability i.e. providing image scrapbooks from recordings as a visual reminder. Findings suggested video-feedback outcomes were positive, suggesting efficacy.

#### Quality appraisal

Pethica and Bigham's (2018) case-study had several shortcomings (meeting 40% quality criteria): observation inter-rater reliability was not reported, the self-rating tool was not validated, and statistical analysis not explained. However, using informal assessment tools seemed clinically appropriate given participant vulnerability.

The adaptations made for people with intellectual disability is a clinical strength, as it accounts for individual needs which could improve efficacy. The study suggests video-feedback may be feasible, when adapted appropriately. However, Pethica and Bigham's (2018) claims video-feedback was efficacious are limited by methodological shortcomings and sample size. Further, research is needed to evaluate video-feedback for parents with intellectual disability particularly, particularly research using experimental and/or randomised design, with a larger sample size. As well as qualitative studies exploring acceptability.

### Caregivers of people with intellectual disability

Various interventions were conducted with caregivers. Of the 6 studies, 3 included parents of children with intellectual disability (up to 14-years-old) (Muddle et al., 2022; Birdsey et al., 2022; Vandesande et al., 2023) and 3 included PCs (of children and adults) (Johnson et al., 2017; Damen et al., 2011; Wingerden et al., 2019; Schuengel et al., 2012). The studies of Johnson et al (2017) and Damen et al (2011) also included people with intellectual disability as participants, as their studies observed specific caregiver-receiver relationships. Five caregiver studies explored efficacy/effectiveness and all six explored acceptability (see Table 3 for further detail).

**Table 3.** Extracted data from caregiver studies.

Author (year)	Study design	Attachment intervention	Total sample size	Summary of participant traits	Data collection methods	Main outcome measures	Qualitative methods and data	Main findings
Birdsey et al. (2022)	Case-series (mixed methods)	Circle of security (COS)	N=6 (n=4 completed, n=2 dropped out)	Parents (pf children aged 5-13, mild-moderate intellectual disability).	Questionnaire (self-report), session transcripts	Parental stress scale; parental acceptance questionnaire; acceptance and action questionnaire; caregiver helplessness questionnaire.	Thematic analysis of session transcripts	**Efficacy:** Quantitative results were mixed: two parents showed no reliable change; one showed increase in stress, psychological inflexibility and acceptance; and one showed reduction in acceptance and mother-helplessness. **Acceptability:** Qualitative themes included “‘*being with’ your child*”, “*being ‘bigger, wiser, stronger, kind*’”, “‘*the COS: exploration and returning to safe hands*’”, “‘*shark music*’”, “‘*rupture and repair*’” and “‘*delight in me*’”.
Damen et al. (2011) & Schuengel et al. (2012)	Case-series (experimental)	Video feedback	N=72 (n=51 in Schuengel et al. (2012)	PCs (study focussed on relationship with 12 SUs, aged 13-54, moderate-severe intellectual disability).	Observations (by researchers), questionnaire (self-report)	Quality of interaction observations (codes: frequency of confirmation used by caregivers, responsiveness and affective mutuality); social validity scale (SVS); adult attachment interview (AII).	N/A	**Efficacy:** Confirmation by caregivers and client initiatives responding to by PCs sig. improved, whilst caregiver initiatives responded to by clients did not. Intervention effects were not sig. moderated by caregiver attachment style. **Acceptability:** All completing the SVS indicated intervention was effective, 63% rated effectiveness as intermediate, none reported negative changes and 61% rated the intervention “workable”.
Johnson et al. (2017)	Mixed-methods (experimental case-series)	Education	N=24 (n=6 dropped out due to changing job)	PCs (study focussed on relationship with 5 SUs, aged 24-52, severe-profound intellectual disability).	Observations (by researchers), field notes, interviews (with PCs)	Positive engagement and relationships observation tool (codes: SU engagement, staff contact interaction mode, relationship processes) created for study; field notes.	Social constructivist inductive analysis of interview transcripts and notes	**Efficacy:** Quantitative findings were variable (observations demonstrated strong effects for one SU, minimal for two SUs and none for two SUs). Findings across SUs varied. **Acceptability:** Qualitative themes included: *“sharing the moment”*, *“recognising individuality”*, *“sharing the message”*, *“training” and “obstacles to changing practice”*. Staff suggested they tried implementing learning but time constraints and capacity were barriers.
Muddle et al. (2022)	Qualitative	COS	N=6	Parents ( of children aged 4-14, moderate-severe intellectual disability).	Interviews and focus groups (of parents)	N/A	Thematic analysis of interview and focus group transcripts	**Acceptability:** Themes included: “*COS concepts are relevant to all children”*, “*parenting children with learning difficulties is different*”, “*COS can create a focus on my child being different and this can be painful*”, “*recommended changes to make COS suitable for parents with learning difficulties*”.
Vandesande et al. (2023)	Mixed-methods	Education	N=16	Parents (of children aged: <10, severe-profound intellectual disability).	Questionnaire (self-report)	Viewing experience questionnaire and knowledge questionnaire (both created for study); perceived maternal parenting self-efficacy scale; daily diaries.	Descriptive illustration of quotes (no formal analysis)	**Efficacy:** No significant changes in self-efficacy or attachment knowledge. All parents reported videos were a good intervention medium and they would recommend to peers.
Wingerden et al. (2019)	RCT	Education	N=100 (11 dropped out) (experimental n=53, WLC n=47)	PCs (adults, mild-moderate intellectual disability).	Questionnaire (self-report)	SVS; knowledge questionnaire (created for study); self-efficacy in nurturing role scale; empathy quotient; interpersonal reactivity index.	N/A	**Efficacy:** Knowledge sig. increased. No effects for empathy and self-efficacy. **Acceptability:** Experimental participants reported small but significant reductions in beliefs around intervention effectiveness at changing behaviour and increases in ease of use.

#### Education

Three papers evaluated education for caregivers. Wingerden et al. (2019) conducted an RCT. Vandesande et al. (2023) and Johnson et al. (2017) used mixed-methods. Johnson et al.'s (2017) study explored whether a relationship model (Johnson et al., 2012) could be translated into an educational intervention to increase PCs’ ability to understand and facilitate relationships for people with intellectual disability.

Both Wingerden et al. (2019) and Vandesande et al. (2023) collected outcomes regarding knowledge, self-efficacy and acceptability. Vandesande et al. (2023) developed a questionnaire to explore acceptability and adapted the parenting self-efficacy scale (for pre-term infants). Whereas Wingerden et al. (2019) used valid and reliable measures of self-efficacy and empathy. Regarding efficacy, Wingerden et al. (2019) found their phone application significantly increased knowledge, however, there were no effects for empathy and self-efficacy. Regarding acceptability, experimental participants reported small but significant reductions in beliefs around intervention effectiveness and increases in beliefs about ease of use. Vandesande et al.'s, (2023) findings also supported acceptability. However, there were no significant changes in self-efficacy or knowledge, suggesting limited efficacy.

Johnson et al. (2017) incorporated observations (of carer interactions with five service users [SUs]), field notes and interviews. Qualitative findings provided insight into staff perceptions of acceptability regarding the training and its implications, including ways the intervention improved relationship practices with SUs. Staff suggested time and capacity were barriers to implementing learning. Overall efficacy findings were variable across SUs. No measure of knowledge was used.

#### Quality appraisal

Wingerden et al.'s (2019) study met 20% of randomised study quality criteria. Although increases in knowledge were positive, practical conclusions cannot be drawn as no behavioural observations were undertaken. It was not possible to determine several quality criteria, possibly due to reporting limitations rather than methodological (i.e. randomisation was not adequately described and it was not clear if all assessors were blinded to intervention during each data collection stage). Experimental condition data was not complete post-intervention, highlighting imperfect adherence. Reasons for incompletion included “lack of time” (n=5) and “I kept forgetting” (n=12) which has acceptability implications. Strengths included RCT methodology and reporting of participant ethnicity (as many included studies omitted this).

Vandesande et al.'s (2023) study maximum quality criteria was only 20% for a mixed methods study. Qualitative rationale and methods were unclear, interpretations were poorly substantiated by data, and confounding variables not considered. All parents completed the videos, supporting feasibility.

Unlike Vandesande et al. (2023), Johnson et al.'s (2017) mixed-method study (incorporating experimental case-series design) met all qualitative quality criteria. However, although they reported using ‘inductive’ analysis, multiple themes were named after elements of the programme, suggesting analysis was not truly data-led. Despite qualitative quality, maximum mixed-method quality criteria was 20%. It was not clear how many invitees responded/declined to take part (or their characteristics) and results did not report reasons for missing data. Regarding measurement, despite good inter-rater reliability for non-occurrences of relationship behaviours, it was poor for occurrences. Furthermore, staff suggested observations were poorly timed. This suggests the observation tool and timing may have been inappropriate. Although mixed-methods was rationalised and integrated to answer the question, interpretation was not drawn from synthesis. A significant strength of the study is assessing practical impact of learning through observations - the only educational study to do so. However, no knowledge measure was used - meaning inferences could not be made about the relationship between increased knowledge and behaviours.

Overall, findings regarding efficacy were varied. Wingerden et al.'s (2019) RCT provided promising evidence for increases in knowledge, despite Vandesande et al.'s (2023) study finding otherwise. Johnson et al.'s (2017) findings regarding efficacy were mixed. All studies could only meet a maximum of 20% quality criteria, due to methodological and/or reporting limitations, meaning findings must be interpreted with caution – despite use of scientifically favoured methods, RCT (particularly) and mixed-methods. Therefore, further high-quality research is required particularly using RCTS and/or practical measures to assess efficacy and acceptability.

#### The circle of security

COS is a parents group aiming to increase secure attachment and sensitivity to their child/children (Mercer, 2015). It uses psychoeducation and therapeutic techniques, including videos and reflection, and also hopes to reduce distressed behaviour and increase parental self-esteem. Evaluations have previously focussed on parents of typically developing children (Muddle et al., 2022), as COS is not intellectual disability specific. Muddle et al. (2022) and Birdsey et al. (2022) evaluated COS for parents of children with intellectual disability. Muddle et al. (2022) collected qualitative data to explore opinions of parents who had completed COS, whilst Birdsey et al. (2022) conducted a mixed-methods case-study.

Themes found by Muddle et al. (2022) suggested parents thought COS was relevant for all children, but adaptations were needed to consider differing attachment and communication needs of children with intellectual disability. This could help to make COS more acceptable and efficacious as some parents found content upsetting. Adaptation suggestions included: more pre-course information; video clips/images of children with intellectual disability, and descriptions of more diverse distressed behaviours. Findings suggest the content of the COS intervention was not acceptable for parents of a child with an intellectual disability.

Birdsey et al. (2022) evaluated efficacy by measuring parental stress, psychological flexibility, acceptance, and helplessness. Efficacy results were mixed. They suggested qualitative data offered explanation for mixed quantitative findings. Similar to Muddle et al. (2022), qualitative findings suggested the intervention was not acceptable, as parents thought COS did not capture differing needs of children with intellectual disability, compared to typically developing children, and how they may need parenting differently. Additionally, group attrition was high (33%).

#### Quality appraisal

Muddle et al. (2022) met 100% qualitative quality criteria. Furthermore, data collection was conducted by researchers who had not facilitated the intervention, which may have reduced bias. Birdsey et al. (2022) met 80% of mixed-method quality criteria. Strengths included collection and interpretation of qualitative data, varied valid and reliable questionnaires, clear rationale and integration of mixed-methods (qualitative findings supported quantitative). However, statistical analysis was not explained.

Both studies suggested COS is not currently acceptable for parents of children with intellectual disability, as current content does not consider their additional parenting, attachment and communication needs. Qualitative findings have practical implications for improving acceptability and efficacy for the population. Parents of children with intellectual disability could help to co-produce and evaluate an adapted COS to make it more appropriate. Evaluation would benefit from addition of observational measures, to assess practical implications. Both studies positively provide literature sharing the parent voice, but are limited by their exploratory nature and small sample size.

#### Video-feedback

Damen et al.'s (2011) study (also reported in Schuengel et al. (2012)), used an AB case-series design to assess video-feedback for PCs, to improve quality of interactions with SUs. Regarding efficacy, quality of interactions was assessed using observations and findings suggested video-feedback led to significant improvements in some aspects. Regarding acceptability, caregivers evaluated perceived intervention effects and ease of use, and were generally positive about intervention.

#### Quality appraisal

Damen et al.'s (2011) study met 80% quality criteria for its methodology. A notable strength is the inter-rater reliability of observations was regularly checked for code drift and retraining provided if agreement was below 80%. Raters were also blinded to the phase of the intervention, reducing possible bias. Furthermore, many PCs partook – suggesting feasibility as a training approach. Quality criteria was not met for low risk of non-response bias as some observation records were incomplete (leading to 11 caregivers being dropped from analysis) and further values were missed to improper completion. Findings suggest video-feedback may be a efficacious and acceptable intervention for PCs. Despite Damen et al.'s (2011) study being of high methodological quality for a case-series, generalisability is limited by small SU sample. Further research is required.

## Discussion

This integrative review aimed to synthesise and appraise quality of research evaluating attachment-based interventions for people with intellectual disability and their caregivers. The review critically appraised 13 studies (15 papers), to explore what types of attachment-based psychological interventions have been implemented for people with intellectual disability across the lifespan, as well as determine whether they are efficacious and acceptable. Evidence was synthesised based on participant groups and interventions for each. Findings support literature highlighting a need for effective attachment-based interventions for people with intellectual disability (Fletcher et al., 2016).

Interventions facilitated directly with people with intellectual disability included education, psychotherapy, technology assisted therapy and video-feedback/VIG (for parents with intellectual disability). Positively preliminary evidence suggests attachment interventions for people with intellectual disability may be efficacious regarding varied attachment-related outcomes. Further research (including experimental and RCT designs) is needed to strengthen evidence and address limited generalisability of current studies. Qualitative or mixed-method research is needed to clarify if participants with intellectual disability find interventions acceptable.

Interventions facilitated directly with caregivers included education, circle of security (COS) and video-feedback**.** Evidence for attachment-based interventions for caregivers of people with intellectual disability is more mixed than studies with people with intellectual disability as direct participants. Caregiver education interventions require further study, including clearly reported RCTs to determine efficacy. COS requires adaptation considering parent feedback and further evaluation. Video-feedback requires further high-quality research to support preliminary efficacy and acceptability evidence.

The current findings add to the evidence base reporting effectiveness of attachment-based interventions for people both with and without intellectual disability (such as neurotypical individuals and those with Autism or developmental delay) and their caregivers (Kennedy et al., 2011; Yaholkoski et al., 2016; Cicchetti et al., 2006; Van Ijzendoorn et al., 2023; Alexander et al., 2023; Hofstra et al., 2023). A number of participants with intellectual disability expressed CB and psychological distress, whilst caregivers identified additional challenges in responding to their attachment needs (Birdsey et al., 2022; Muddle et al., 2022; Sterkenburg et al., 2008a; Sterkenburg et al., 2008b; Hoffman et al., 2019; Jonker et al., 2015; McInnis, 2016). This supports literature suggesting people with intellectual disability experience additional attachment-related difficulties and reinforces that standard attachment-based interventions and measures require appropriate adaptation and sensitivity for the population (Giltaij et al., 2015; Schuengel and Janssen, 2006; Skelly, 2016; De Schipper and Schuengel, 2010; Walker et al., 2016).

Given people with intellectual disability often require additional support throughout their lifespan, including from PCs/supported living and residential care (Fletcher et al., 2016), it is promising that interventions took place across the lifespan, within an array of settings and considered different types of attachment-relationships.

### Strengths and limitations of literature

Researchers and participants undertaking the current studies, have positively contributed to an important but sparse field, which can be challenging to research (due to sample heterogeneity, as well as additional vulnerabilities, communication and measurement considerations).

Several included studies had high quality methodology for their type of research as assessed by the MMAT (Pearson et al., 2019; Sterkenburg et al., 2008b; Sterkenburg et al., 2008a; Birdsey et al., 2022; Muddle et al., 2022; Damen et al., 2011; Hong et al., 2018), but were limited by other factors. Although case-studies, case-series, qualitative and non-randomised studies can provide important preliminary evidence regarding intervention efficacy and acceptability, they are not as informative as RCTs which are a gold standard method of research (Hariton and Locascio, 2018). Only one RCT was included in the current study, and it was limited by methodological or reporting shortcomings (Wingerden et al., 2019).

Despite case-studies and case-series not lending well to generalisable research, they lend well to clinical work and may reflect the reality of interventions people with intellectual disability typically receive from services (Pethica and Bigham, 2018; McInnis, 2016). Studies reporting adaptations made to support individual needs of participants provide helpful ideas which could support individualised/person-centred care, based on a person’s cognitive abilities, i.e. using visual scales for measures and scrapbooks/take-away material to reinforce learning (Pethica and Bigham, 2018). Only one study in the current review explored intervention acceptability for participants with an intellectual disability (Jonker et al., 2015), this is a significant limitation of the current literature and requires further attention to ensure people with intellectual disability are satisfied and comfortable with the interventions they receive.

As highlighted in previous literature, measurement of interventions was inconsistent (Hamadi and Fletcher, 2021). Although many studies used valid and reliable measures, the use across papers was varied. This is not surprising given the broad scope of attachment-based interventions included within the study, but may in part be due to the lack of validated attachment measures for people with intellectual disability (Walker et al., 2016). Measures used to assess interventions included those for distressed behaviour, psychological distress/symptoms, self-efficacy, self-esteem, parental acceptance, QoL, anxiety, observations of interactions, parental stress and knowledge. Although these factors are all relevant to attachment, lack of consistent measurement across studies means it can be difficult to compare and synthesise findings.

Further research evaluating attachment-based interventions using a standardised combination of measures such as questionnaires and practical observations tools (exploring attachment relationships as well as other attachment-relevant behaviours and psychological outcomes), could help to strengthen the field by demonstrating both theoretical and practical implications whilst supporting comparisons across research. Further investigation is needed to develop and validate attachment-related measures for people with intellectual disability, to encourage consistent and standardised use across research.

### Additional implications

It is evident from the current studies that researchers and clinicians agree attachment theory is particularly important to consider for people with intellectual disability (BPS, 2017). Further high-quality research, with appropriate sample size, is needed to evaluate efficacity and acceptability of the discussed interventions. Such research will help to inform clinical provision within intellectual disability services.

Qualitative findings suggest, clinicians should be sensitive to differing needs of children with intellectual disability and their parents, and adapt interventions and/or information accordingly (i.e. including images/videos of people with intellectual disability in materials/examples) (Birdsey et al., 2022; Muddle et al., 2022). Clinicians providing attachment-based interventions for people with intellectual disability and caregivers, should robustly evaluate their work and publish findings, to help add to the limited evidence base. This should include reporting qualitative feedback regarding acceptability for people with intellectual disability.

### Strengths and limitations of the current review

A strength of the current integrative synthesis is being the first to evaluate attachment-based interventions for people with intellectual disability across the lifespan. Although also considered a strength of the current review in its ability to provide a broad overview of the literature, by exploring a broad conceptualisation of attachment-based intervention, as well as quantitative and qualitative methodologies, it was difficult to directly compare and synthesise studies. The MMAT is a useful appraisal tool for mixed study integrative reviews, as it can evaluate studies with different methodologies (Hong et al., 2018). However, it has been suggested MMAT criteria can be more difficult to judge than other appraisal tools as they focus on quality of method not reporting quality (Hong and Pluye, 2019; Hong et al., 2018). The current lead author and independent reviewer met to discuss any tool criteria and guidance which needed further clarity. Furthermore, by nature of the MMAT tool grouping and appraising different methodologies with different criteria, it does not highlight or specify which type of methodologies are of strongest scientific value, nor use methodologically specific assessment criteria for all types of studies. Given six of the 13 studies in the current review used experimental case-study or series design, it could be helpful for a future review to explore and appraise these types of studies specifically.

For appraisal items marked “can’t tell” the current authors did not contact authors of assessed papers to determine whether criteria were met but not adequately reported. Therefore, quality of such studies may be higher than assessed in the current review.

## Conclusion

This systematic integrative review synthesised findings from 13 studies (15 papers) each evaluating an attachment-based intervention for people with intellectual disability and/or their caregivers. Findings were divided by intervention participants. Attachment-based interventions included education, psychotherapy, technology assisted therapy for SA, video-feedback/VIG, and COS. Overall, evidence regarding efficacy and acceptability of interventions was promising and requires further high-quality research (lending itself to generalisability) to build on preliminary findings.

## Appendix

**Appendix 1. Mixed Methods Appraisal Tool Quality Questions and Criteria.** Mixed Methods Appraisal Tool Quality Questions and Criteria.

Category of study designs	Methodological quality criteria
Screening questions (for all types)	S1. Are there clear research questions? S2. Do the collected data allow to address the research questions?
1. Qualitative	1.1. Is the qualitative approach appropriate to answer the research question? 1.2. Are the qualitative data collection methods adequate to address the research question? 1.3. Are the findings adequately derived from the data? 1.4. Is the interpretation of results sufficiently substantiated by data? 1.5. Is there coherence between qualitative data sources, collection, analysis and interpretation?
2. Quantitative randomized controlledtrials	2.1. Is randomization appropriately performed? 2.2. Are the groups comparable at baseline? 2.3. Are there complete outcome data? 2.4. Are outcome assessors blinded to the intervention provided? 2.5 Did the participants adhere to the assigned intervention?
3. Quantitative non-randomized	3.1. Are the participants representative of the target population? 3.2. Are measurements appropriate regarding both the outcome and intervention (or exposure)? 3.3. Are there complete outcome data? 3.4. Are the confounders accounted for in the design and analysis? 3.5. During the study period, is the intervention administered (or exposure occurred) as intended?
4. Quantitative descriptive	4.1. Is the sampling strategy relevant to address the research question? 4.2. Is the sample representative of the target population? 4.3. Are the measurements appropriate? 4.4. Is the risk of nonresponse bias low? 4.5. Is the statistical analysis appropriate to answer the research question?
5. Mixed methods	5.1. Is there an adequate rationale for using a mixed methods design to address the research question? 5.2. Are the different components of the study effectively integrated to answer the research question? 5.3. Are the outputs of the integration of qualitative and quantitative components adequately interpreted? 5.4. Are divergences and inconsistencies between quantitative and qualitative results adequately addressed? 5.5. Do the different components of the study adhere to the quality criteria of each tradition of the methods involved?

### Appendix 2: Search Terms

The search terms aimed to capture intellectual disability and attachment-based intervention studies. Outdated terms for intellectual disability were included to ensure all relevant papers were found. Their use is not endorsed by the author.

1. “Intellectual disab*” OR “learning disab*” OR “learning difficult*” OR “intellectual difficult*” OR “intellectual impair*” OR “learning impair*” OR “developmental disorder” OR “developmental disab*” OR “learning handicap” OR “mental* handicap*” OR “intellectual* handicap*” OR “mental retard*”
2. attachment*
3. Training OR intervention OR therapy OR “video interaction guidance” OR “video feedback”
